# Structural, Thermal, Pasting and Digestion Properties of Starches from Developing Root Tubers of Sweet Potato

**DOI:** 10.3390/foods13071103

**Published:** 2024-04-03

**Authors:** Hao Wang, Yuanhao Feng, Ke Guo, Laiquan Shi, Xin Xu, Cunxu Wei

**Affiliations:** 1 Key Laboratory of Crop Genetics and Physiology of Jiangsu Province/Joint International Research Laboratory of Agriculture & Agri-Product Safety of the Ministry of Education, Yangzhou University, Yangzhou 225009, China; mx120211052@yzu.edu.cn (H.W.); mz120221770@yzu.edu.cn (Y.F.); 18115657147@163.com (K.G.); dx120220208@yzu.edu.cn (L.S.); mx120211067@yzu.edu.cn (X.X.); 2 Co-Innovation Center for Modern Production Technology of Grain Crops of Jiangsu Province/Jiangsu Key Laboratory of Crop Genomics and Molecular Breeding, Yangzhou University, Yangzhou 225009, China; 3 Institute of Food Crops, Jiangsu Academy of Agricultural Sciences, Nanjing 210014, China

**Keywords:** sweet potato, developing root tuber, starch, physicochemical properties

## Abstract

Three sweet potato varieties with white-, yellow- and purple-fleshed root tubers were harvested at 100, 120, 140 and 160 days after planting (DAP). Their starch structural, thermal, pasting and digestion properties were measured to reveal the influences of harvesting dates on the physicochemical properties of sweet potato root tuber starch. Though starches from different varieties displayed some differences in physicochemical properties due to their different genetic backgrounds, they were influenced by harvesting date in similar ways. Starches isolated from root tubers at 100 and 160 DAP exhibited lower granule sizes than those at 120 and 140 DAP. The amylose content was higher in root tubers at 100 and 120 DAP than at 140 and 160 DAP. Starches from root tubers at 100 DAP exhibited C_A_-type X-ray diffraction patterns, and then the B-type crystallinity gradually increased at later harvesting dates. The different harvesting dates had no significant effects on the short-ranged ordered structure and lamellar thickness of starch, but the lamellar peak intensity decreased significantly at later harvesting dates. Starch had a lower gelatinization temperature and a wider gelatinization temperature range in root tubers at 140 and 160 DAP than at 100 and 120 DAP. The higher peak viscosity and lower pasting temperature were associated with the late harvesting date. The digestion of starch had slight differences among root tubers at different harvesting dates. The harvesting dates of root tubers played more important roles in starch properties than the variety. This study would be helpful for breeders, farmers and sweet potato starch users.

## 1. Introduction

Native (or raw) starches from plant sources are semi-crystalline granules, mainly consisting of amylose and amylopectin. The shapes and sizes of starch granules vary according to their botanical sources. The different shapes include spheres, polygons, irregular tubules and ellipsoids, and the sizes can range from 0.1 to 100 μm [1,2]. Amylose, a mainly linear and slightly branched glucose polymer, constitutes 5~35% of most natural starches and has a major influence over the starch properties in foods [3]. Amylopectin, a highly branched glucose polymer, has approximately 95% of α-(1,4) glycosidic bonds and about 5% of α-(1,6) glycosidic bonds. Its branch-chain lengths and arrangements are important factors in determining the semi-crystalline structure [4]. The shape, size, amylose content and amylopectin arrangement of native starches depend on their botany sources, and play critical roles in starch properties and applications in food and non-food industries [1,2,3,4,5]. Starch is obtained mainly from conventional sources such as maize, rice, potato and cassava. With the increasing demand of starch in food and non-food industries, nonconventional and underutilized starch sources of roots, root tubers, tubers, rhizomes, bulbs, fruits and pseudocereal have been reported in recent years [1,5,6,7,8,9,10].

Sweet potato (*Ipomoea batatas*), an important root tuber crop, provides food and energy for people, especially in Asia and Africa [11,12]. Sweet potato has white-, purple- and yellow-fleshed root tubers, which contain over 60%, 53% and 45% starch in dry weight, respectively [11,12,13]. Sweet potato starch is widely used to produce noodles and vermicelli, and is also a main raw material in both food and non-food industries [14,15]. Therefore, many studies have reported the physicochemical properties of sweet potato starch [13,16,17,18,19,20,21,22,23,24,25,26,27].

Sweet potato is widely planted in tropical, subtropical and temperate countries. Under natural environments and in different countries, the planting and harvesting dates of sweet potato vary due to the growth temperature limitation. Though sweet potato root tuber is a vegetative storage tissue and can keep on growing and accumulating starch under a suitable growth environment, it is usually harvested during development. The study of Noda et al. [16] showed that both planting and harvesting dates have profound effects on the granule size, crystalline structure, pasting properties, thermal properties and hydrolysis rate of sweet potato starch, but do not affect the amylose content. The growth temperature is considered to be a main factor that influences starch properties in sweet potato. Some researchers plant sweet potatoes in a temperature-controlled growth environment, investigating the effects of the growth temperature on starch properties [17,23]. The crystalline structure of sweet potato starch is very sensitive to the growth temperature. B-type crystallinity gradually forms and accumulates with decreasing growth temperatures, and A-type crystallinity forms and accumulates with increasing growth temperatures [17,23]. From practical production, it is more important to investigate the effects of the natural growth environment on the properties of sweet potato starch. dos Santos et al. [27] reported that, when compared to a dry season with low soil temperatures, the rainy season with high soil temperatures increases the ordered structure, gelatinization temperature and resistant starch content of sweet potato starch. The production of sweet potato is contributed to mostly by China [11]. In China, cuttings of sweet potatoes can be planted from April, and root tubers are harvested from September to November. Therefore, it is important to investigate the effects of harvesting dates on starch properties in sweet potato.

In this study, three popular sweet potato varieties, Ningzishu 1 (NZS1) with purple-fleshed root tuber, Sushu 16 (SS16) with yellow-fleshed root tuber and Sushu 28 (SS28) with white-fleshed root tuber, were planted in a field with the same environment. Their root tubers were harvested at different days after planting (DAP). Our objective is to study the characteristics of starches from the developing root tubers of sweet potato, revealing the effects of harvesting dates on starch properties. The results would be helpful for breeders, farmers and sweet potato starch users.

## 2. Materials and Methods

### 2.1. Plant Materials

**Figure 1:**
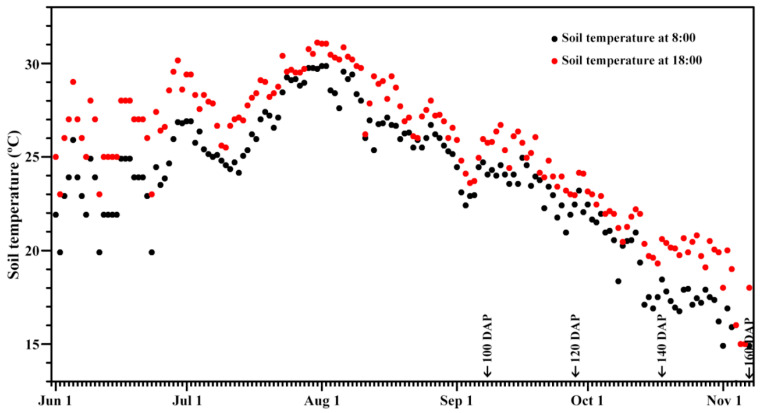
Soil temperatures during sweet potato growth.

Three popular sweet potato varieties, Ningzishu 1 (NZS1) with purple-fleshed root tuber, Sushu 16 (SS16) with yellow-fleshed root tuber and Sushu 28 (SS28) with white-fleshed root tuber, were planted in the experimental field of Yangzhou University in 2020. The field management and fertilizer treatment were the same among the three varieties. The cuttings were planted on May 31, and the root tubers were harvested at 100, 120, 140 and 160 DAP. During sweet potato growth, the daily soil temperature at 10 cm underground was measured at 8:00 and 18:00 (Figure 1).

### 2.2. Isolation of Starches from Root Tubers

The starch was isolated following our previous procedures [13]. Briefly, fresh root tuber was washed and chopped into small pieces, and then homogenized in water using a household kitchen mixer. The sample was filtered through 4 layers of cheesecloth and 100-, 200- and 300-mesh sieves. Starch and water slurry stood for over 5 h. Precipitated starch was washed with water until a clear supernatant was evident. Finally, starch was washed many times with anhydrous ethanol until a clear supernatant was observed and then dried at 40 °C.

### 2.3. Granule Shapes and Sizes of Starch Granules

Starch (2 mg) in 1 mL 50% glycerol aqueous solution was observed and photographed under a polarized microscope (BX53, Olympus, Tokyo, Japan) under both normal and polarized light, following the method of Ren et al. [9]. For size analysis, starch suspension in water was analyzed with a laser diffraction particle size analyzer (Mastersizer 2000, Malvern, Worcestershire, UK). During analysis, the sample was stirred at 2000 rpm and the opacity was controlled between 10% and 11%. The volume-weighted diameter d(0.5) and D[4,3] were chosen as the granule size. This analysis was repeated thrice.

### 2.4. Iodine Colorimetry Analysis of Starch

The iodine colorimetry of starch followed our previous method [13]. Starch (10 mg) was dissolved in 5 mL dimethyl sulfoxide containing 10% (*v*/*v*) 6.0 M urea at 95 °C and then cooled to room temperature. The sample (1 mL) was colored with 1 mL iodine solution (0.2% I_2_, 2% KI) in 50 mL solution, and then scanned from 400 to 900 nm with a spectrophotometer (BioMate 3S, Thermo Scientific, Chino, CA, USA). The amylose content was calculated using OD_620_ with a reference of maize amylopectin and potato amylose. The experiments were repeated thrice.

### 2.5. Crystalline Structure Analysis of Starch

Starch was detected with an X-ray powder diffractometer (D8, Bruker, Karlsruhe, Germany). Starch treatment and testing conditions were all the same as that of Guo et al. [13]. The relative crystallinity (RC) is the percentage of the crystalline peak area to the total diffraction area over the diffraction angle 4−30° 2θ. The experiments were repeated twice.

### 2.6. Short-Ranged Ordered Structure Analysis of Starch

The starch–water slurry (4:6, *w*:*w*) was scanned from 800 to 4000 cm^−1^ using a Fourier transform infrared (FTIR) spectrometer (7000, Varian, Santa Clara, CA, USA) with an attenuated total reflectance (ATR) cell, following our previous method [13]. The experiments were repeated twice.

### 2.7. Lamellar Structure Analysis of Starch

The starch–water suspension (1:10, *w*:*w*) was centrifuged at 3000× *g* for 5 min, and the starch precipitation was analyzed using a small-angle X-ray scattering (SAXS) analysis system (NanoStar, Bruker, Karlsruhe, Germany). The starch treatment, testing condition and lamellar parameter measurement were all the same as the methods and procedures of Guo et al. [23]. In order to compare the lamellar peak intensities among different starches, the intensities at scattering vector 0.2 Å^−1^ were all normalized to 20. The experiments were repeated twice.

### 2.8. Thermal Property Analysis of Starch

Starch (5 mg) was weighed in an aluminum pan, and water (15 μL) was added and sealed. The sample was equilibrated for over 2 h, and then heated with a differential scanning calorimetry (DSC) (DSC 200-F3, Netzsch, Selb, Germany) at 10 °C/min. The gelatinization onset temperature (To), peak temperature (Tp), conclusion temperature (Tc) and enthalpy (∆H) were evaluated with a thermogram using the analysis software from the DSC manufacturer. The experiments were repeated thrice.

### 2.9. Pasting Property Analysis of Starch

The starch (2.5 g) and water (25 mL) slurry was maintained at 50 °C for 1 min, heated from 50 to 95 °C for 3.75 min, maintained at 95 °C for 2.5 min, cooled from 95 to 50 °C for 3.75 min and maintained at 50 °C for 1.4 min using a rapid visco analyzer (RVA) (3D, Newport Scientific, Warriewood, Australia). The sample was mixed at 160 rpm during analysis. The experiments were repeated twice.

### 2.10. Digestion Analysis of Starch

The gelatinized starch was prepared via heating the 1% (*w*/*w*) starch aqueous solution in a ThermoMixer at 1000 rpm for 12 min at 98 °C. The digestion of native starch and gelatinized starch was investigated completely following our previous method [13]. Briefly, starch was hydrolyzed by both porcine pancreatic α-amylase (PPA) (Sigma, A3176, St. Louis, MO, USA) and *Aspergillus niger* amyloglucosidase (AAG) (Megazyme, E-AMGDF, Bray, Ireland) at 37 °C. The produced glucose determined by the D-glucose Assay Kit (Megazyme, K-GLUC, Bray, Ireland) was converted to degraded starch. The total starch was determined with the Total Starch Assay Kit (K-TSTA, Megazyme, Bray, Ireland). The percentage of degraded starch to total starch was determined. The experiments were repeated thrice.

### 2.11. Principal Component Analysis

Minitab 16 (IBM Company, Chicago, IL, USA) was used for PCA based on the starch characteristic data with a significance of normal distribution over 0.05. The score and loading plots for the first two components were given.

### 2.12. Statistical Analysis

The significance of data normal distribution and the significant difference of data were investigated using the Shapiro–Wilk test and Tukey’s one-way ANOVA of SPSS 16.0, respectively.

## 3. Results and Discussion

### 3.1. Granule Shape and Size of Starch

**Figure 2:**
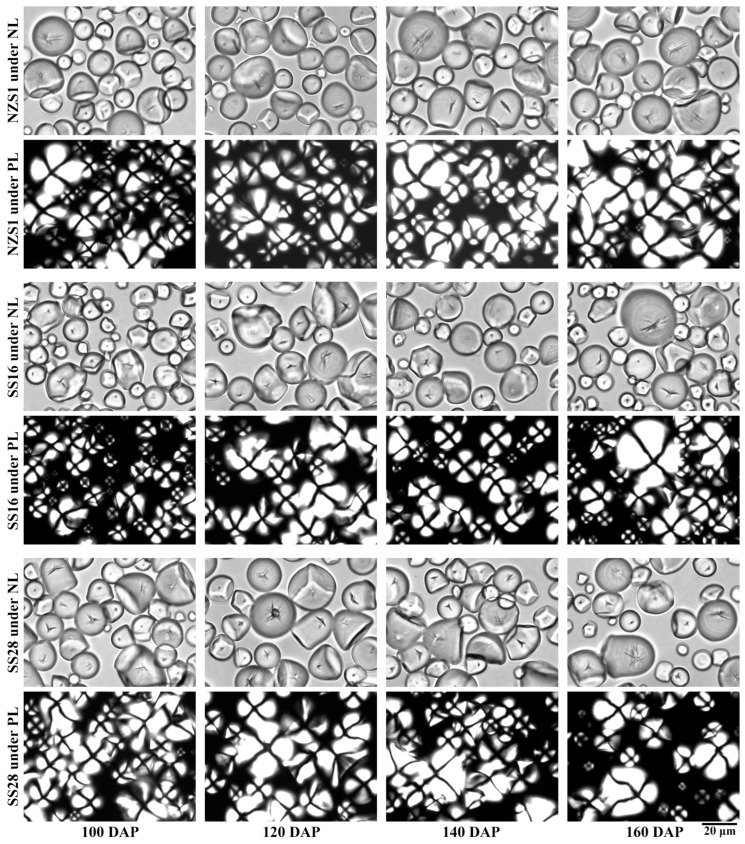
Granule shapes of different starches under normal light (NL) and polarized light (PL).

**Figure 3:**
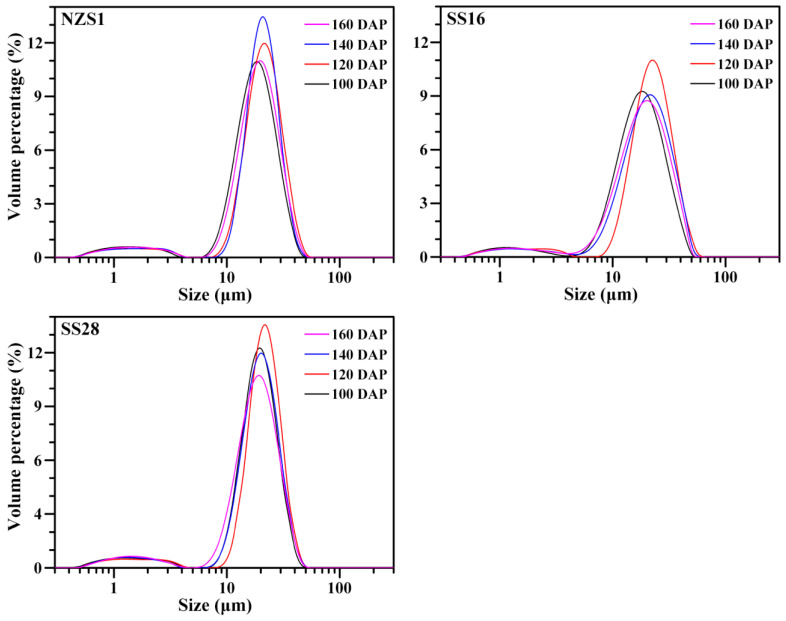
Granule size distributions of starches from the developing root tubers of sweet potatoes.

Starch isolated from developing root tubers exhibited some round, polygonal, oval and semi-oval shapes, and they had different granule sizes under normal light. Under polarized light, starch granules showed central “Maltese crosses”. Starches from the root tubers of three sweet potato varieties at 100, 120, 140 and 160 DAP displayed no significant differences in terms of granule shape (Figure 2). Similar shapes of starch granules have also been reported in sweet potato root tubers [23]. Starch size is an important physicochemical property, influencing the functional properties and applications of starch [25]. Starches from the developing root tubers of three sweet potato varieties at 100, 120, 140 and 160 DAP had similar granule size distributions, ranging from 8 to 40 μm (Figure 3). The granule diameter d(0.5) and D[4,3] ranged from 16.3 to 20.5 μm and from 17.0 to 21.1 μm, respectively, among starches from the developing root tubers of three sweet potato varieties (Table 1). The size distribution agreed with previous reports [12,25]. Though starch size is attributed to botany sources, the growth environment, especially the temperature, can influence starch size [1,2]. In this study, starches from three sweet potato varieties all exhibited lower granule sizes at 100 and 160 DAP than at 120 and 140 DAP (Table 1). The soil temperature was over 27 °C during the time between 15 July and 7 September (100 DAP), between 27 and 21 °C from 7 September to 17 October (140 DAP) and below 21 °C from 17 October to 6 November (160 DAP) (Figure 1). Noda et al. [16] reported that the starch granule size increases with a later harvesting date; however, Noda et al. [17] also reported that sweet potato starch increases in granule size under soil temperatures between 15 and 27 °C, but then decreases in size after 27 °C. Guo et al. [23] found that the starch granule size markedly increases when the soil temperature increases from 21 to 25 °C, but maintains a similar granule size at 25 and 28 °C. Therefore, high and low soil temperatures have adverse effects on the size of starch, and suitable soil temperatures, from about 21 to 27 °C, are beneficial for the granular growth of sweet potato starch.

### 3.2. Iodine Absorption and Amylose Content of Starch

**Figure 4:**
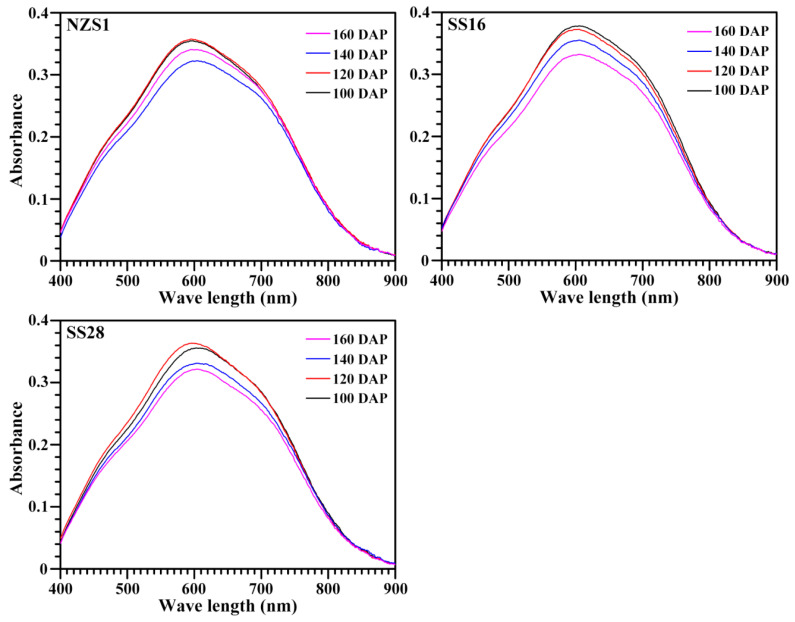
Iodine absorption spectra of starches from developing root tubers of sweet potatoes.

The starch–iodine absorption spectrum can reflect the starch components [28]. The starches from three sweet potato varieties all exhibited similar iodine absorption spectra at 100 and 120 DAP, which were significantly stronger than at 140 and 160 DAP (Figure 4). The iodine blue value at 680 nm (OD_680_) is usually used to reflect the binding ability of starch and iodine, and the absorbance ratio of the starch–iodine complex at 620 to 550 nm (OD_620/550_) can indicate the content of the longer chain segments in starch [28]. The OD_680_ ranged from 0.273 to 0.328 among the starches from the developing root tubers of three sweet potato varieties. The OD_620/550_ is similar among these starches; however, the amylose content at 100 and 120 DAP was higher than at 140 and 160 DAP (Table 1). Noda et al. [17] reported that the amylose content determined by iodine colorimetry decreases significantly in sweet potato with decreasing soil temperatures, agreeing with the present study.

### 3.3. Crystalline Structure of Starch

**Figure 5:**
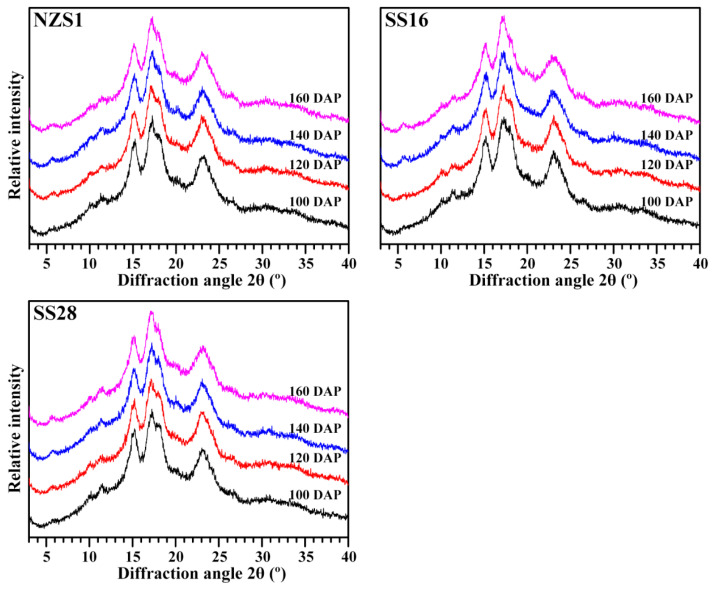
XRD patterns of starches from the developing root tubers of sweet potatoes.

Starches from different plant resources are usually divided into A-, B- and C-type crystalline starch according to their X-ray diffraction (XRD) patterns [29,30]. In fact, amylopectin branch-chains form only A- and B-type crystallinity. A-type starch containing only A-type crystallinity exhibits a typical diffraction doublet at 2θ 17° and 18° and two strong diffraction peaks at 2θ 15° and 23° in its XRD pattern. B-type starch containing only B-type crystallinity has one strong diffraction peak at 2θ 17°, three small peaks at 2θ 15°, 22° and 24° and a B-type characteristic peak at 2θ 5.6°. C-type starch containing both A- and B-type crystallinities has a mixture diffraction peaks of A- and B-type starches. C-type starches can be further divided into C_A_-, C_C_- and C_B_-type according to the content ratio of A- and B-type crystallinity from high to low. The XRD pattern of the C_C_-type starch exhibits a strong singlet at 2θ 17° and 23° and two small peaks at 2θ 5.6° and 15°. Compared with the C_C_-type XRD pattern, the C_A_-type XRD pattern has a shoulder peak at 2θ 18° and the C_B_-type XRD pattern has two shoulder peaks at 2θ 22° and 24° [29,30]. Starches from three sweet potato varieties at 100 DAP all exhibited C_A_-type XRD patterns with an obvious diffraction shoulder peak at 2θ 18°. With the development of root tubers, the shoulder peak at 2θ 18° and the peaks at 2θ 15° and 23° gradually became weak, and the peaks at 2θ 5.6° became stronger, indicating that the B-type crystallinity increased, and the starch became C_C_-type (Figure 5). A-, C_A_-, C_C_- and C_B_-type XRD patterns are all reported in starches from sweet potato root tubers, and the complicated crystalline polymorphic structure is due to the fact that the crystalline structure formation is sensitive to the growth conditions of the root tuber, especially the soil temperature [12,16,18,19,23,27]. Noda et al. [16] detected that sweet potato starch exhibits a C-type XRD pattern, tending to shift to the A-type at the early harvesting date and then the B-type at the late planting or harvesting date. Genkina et al. [18,19] detected that sweet potatoes have A-type starches at soil temperatures between 27 and 33 °C, gradually form and accumulate B-type crystallinity with decreasing soil temperatures and have C_B_-type starches with a soil temperature of 15 °C. Guo et al. [23] detected that sweet potato starch exhibits a C_C_-type XRD pattern at 21 °C, C_A_-type at 25 °C and closest to A-type at 28 °C. dos Santos et al. [27] detected that sweet potato starch shows an A-type XRD pattern in the rainy season with high soil temperatures and a C_A_-type in the dry season with low soil temperatures. In this research, the soil temperature gradually decreased from 100 to 160 DAP, agreeing with previous reports that low soil temperature is favorable to form the B-type crystallinity. The relative crystallinity (RC) of starch ranged from 21.6% to 26.7% among the developing root tubers of three sweet potato varieties (Table 2). The RC is influenced by many factors, including the granule size, amylose content, amylopectin structure and crystallinity type [13].

### 3.4. Short-Ranged Ordered Structure of Starch

**Figure 6:**
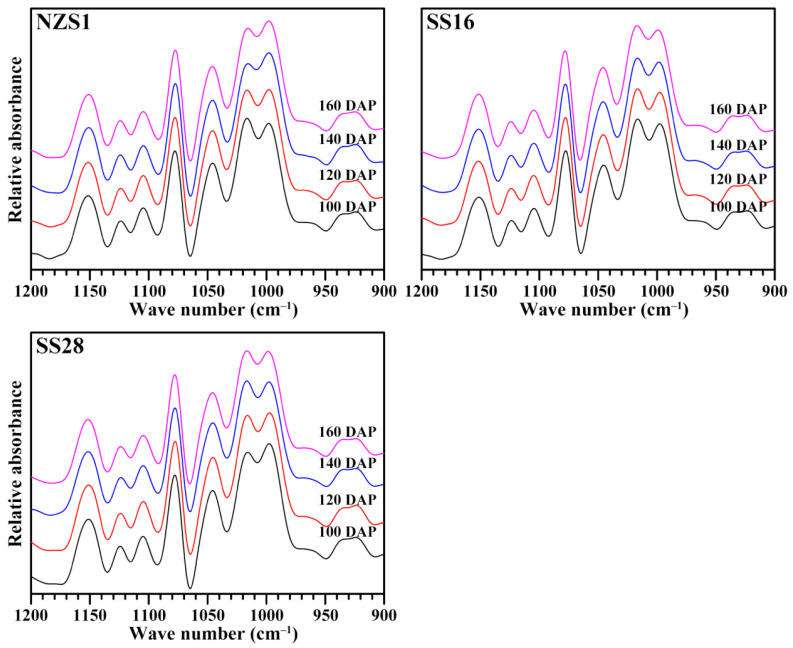
FTIR spectra of starches from the developing root tubers of sweet potatoes.

The attenuated total reflectance-Fourier transform infrared (ATR-FTIR) spectrometer can analyze the short-ranged ordered structure at the external region of the starch granules [31]. Starches from the root tubers of three sweet potato varieties had similar FTIR spectra (Figure 6). The FTIR spectrum is usually converted to an IR ratio of absorbance at 1045 to 1022 cm^−1^ (IR_1045/1022_) and at 1022 to 995 cm^−1^ (IR_1022/995_) in order to quantitatively compare the ordered degree of starch. The former reflects the ordered degree, and the latter can be used as a measure of the proportion of amorphous to ordered carbohydrate structures within the starch [31]. Starches from the developing root tubers of three sweet potato varieties had similar IR_1045/1022_ between 0.643 and 0.702 and IR_1022/995_ between 0.914 and 1.056 (Table 2), indicating that different harvesting dates had no significant effects on the short-ranged ordered structure at the external region of the starch granule.

### 3.5. Lamellar Structure of Starch

**Figure 7:**
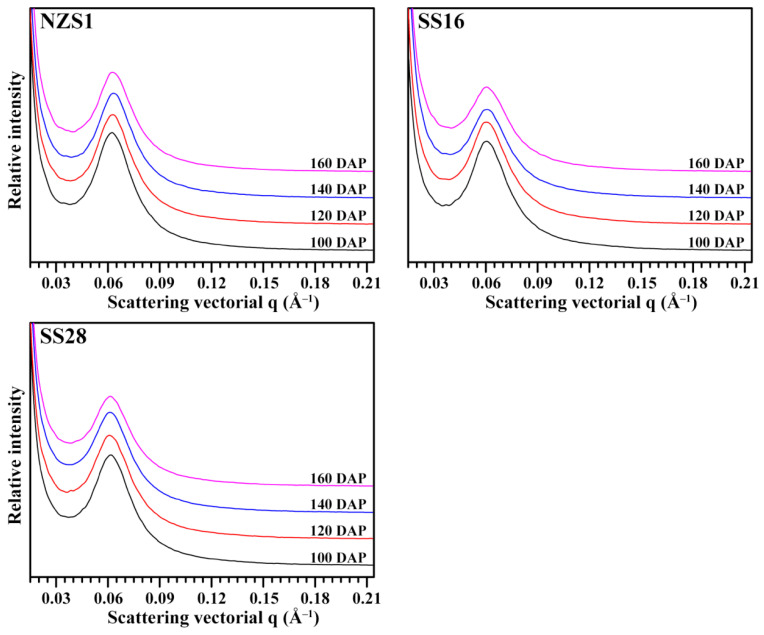
SAXS profiles of starches from the developing root tubers of sweet potatoes.

The small-angle X-ray scattering (SAXS) profiles of starches were presented in Figure 7. The well-resolved main scattering peak, at about 0.06 Å^−1^, is thought to arise from the periodic arrangement of the alternating crystalline and amorphous lamellae of amylopectin, and corresponds to the lamellar thickness. The peak position may differ for starch originating from different plant sources, while the peak intensity (PI) or peak area mainly reflects the electron density difference between the crystalline and amorphous lamellae of starch, and depends mainly on the degree of ordering in semicrystalline regions [32]. The PI, maximum peak position (S_Max_) and lamellar repeat distance (D) of starches from developing root tubers were presented in Table 2. Though S_Max_ and D did not significantly change among starches from the developing root tubers of three sweet potato varieties, the PI gradually decreased significantly with the root tuber development from 100 to 160 DAP. The PI of starch from 100 to 160 DAP decreased from 467.8 to 394 in NZS1, from 425.9 to 293.8 in SS16 and from 421.5 to 324.5 in SS28 (Table 2). Guo et al. [23] reported that starches from sweet potato root tubers with constant soil temperatures of 28, 25 and 21 °C had lamellar PIs of 591.3, 529.5 and 433.4, respectively. The B-type starch exhibits significantly lower lamellar PI than the A-type starch [9]. In this study, starch gradually accumulate more B-type crystallinity in root tubers from 100 to 160 DAP, which could be one of the predominant reasons for the decrease in the lamellar PI during root tuber development.

### 3.6. Thermal Properties of Starch

**Figure 8:**
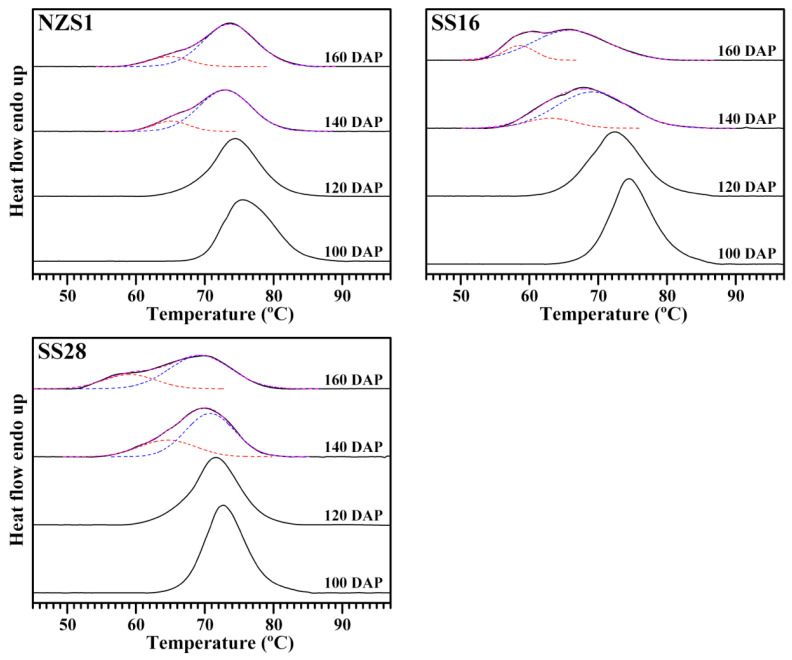
DSC thermograms of starches from the developing root tubers of sweet potatoes. The black curve is the original thermogram following the baseline correction, the red and blue dash curves are the fitting peaks and the magenta dash curve is the fitting thermogram.

The differential scanning calorimetry (DSC) thermograms of starches were showed in Figure 8, and their thermal property parameters were summarized in Table 3. The gelatinization onset (To), peak (Tp) and conclusion temperature (Tc) ranged from 51.3 to 69.8 °C, from 66.0 to 75.6 °C and from 78.2 to 84.1 °C, respectively, and the gelatinization temperature (∆T) and enthalpy (∆H) ranged from 12.3 to 26.9 °C and from 8.45 to 11.84 J/g, respectively, among the starches from the developing root tubers of three sweet potato varieties (Table 3). The single gelatinization peak with a high gelatinization temperature was observed in the starches from the root tubers of three sweet potato varieties at 100 and 120 DAP. The obvious second gelatinization peak with a low gelatinization temperature was detected in root tuber starches at 140 and 160 DAP, thus leading to the decrease in the gelatinization temperature and the increase in the gelatinization temperature range (Figure 8, Table 3). Noda et al. [16] also reported that sweet potato starch has a higher gelatinization temperature at an early harvesting date than at a late harvesting date. High soil temperatures significantly increase the starch gelatinization temperature in sweet potatoes [17]. Many references reported that sweet potato starches have very wide ∆T when root tubers are subjected to both high and low temperature growth stages [19,22,25]. Guo et al. [22] reported that sweet potato root tubers simultaneously have A-type starch granules with high gelatinization temperatures, B-type starch granules with low gelatinization temperatures and C-type starch granules with intermediate gelatinization temperatures, resulting in a very wide DSC thermal peak. In this research, starches from root tubers at 100 and 120 DAP had high gelatinization temperatures due to the starch formation occurring at high soil temperatures, and the starch from the root tubers at 140 and 160 DAP had wide gelatinization peaks due to the B-type crystallinity accumulation. The thermal properties of starches agreed with their crystalline structure variation during root tuber growth.

### 3.7. Pasting Properties of Starch

**Figure 9:**
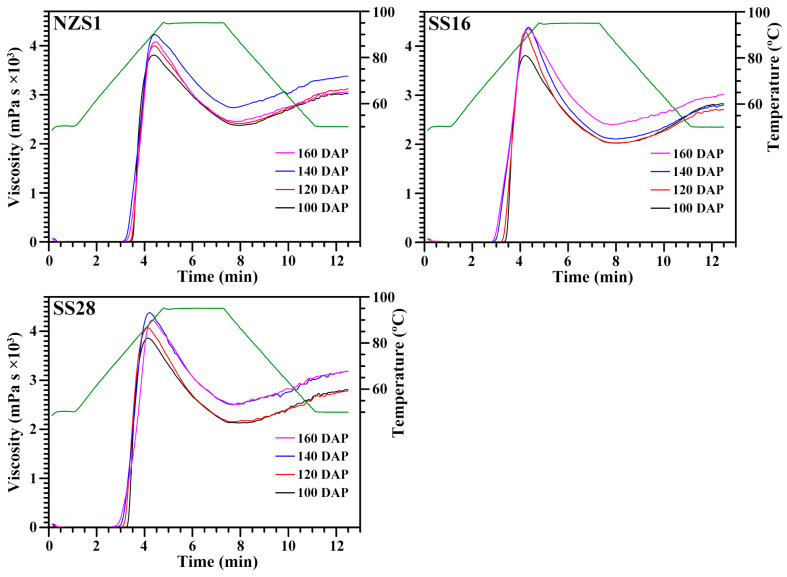
RVA profiles of starches from the developing root tubers of sweet potatoes.

The RVA profiles of starches were presented in Figure 9. During analysis, the starch–water solution was subjected to a programmed heating and cooling treatment. Starch absorbs water and swells when heated, resulting in a significant increase in viscosity above the pasting temperature and eventually reaching a peak viscosity. With continued heating, starch granules rupture and allow amylose and amylopectin to leak into the surrounding solution, thus resulting in a reduction in viscosity. As the temperature decreases, the intermolecular distances are shortened, causing the mixture to form a gel and the viscosity to increase again [33]. In order to quantitatively compare the viscosity variation, seven primary parameters, including peak viscosity (PV), hot viscosity (HV), final viscosity (FV), breakdown viscosity (BV), setback viscosity (SV), peak time (P_Time_) and pasting temperature (P_Temp_), can be obtained from the RVA profiles. The PV is the first peak paste viscosity following gelatinization; HV is the paste viscosity at trough after PV; FV is the paste viscosity at the end of the final holding period; BV (PV − HV) is the decrease in viscosity during cooking; SV (FV − HV) is the increase in viscosity during the cooling period; P_Time_ is the time required to reach PV; P_Temp_ is the temperature of the initial viscosity increase [34]. PV reflects the ability of starch to bind water via hydrogen bonds; BV can evaluate the starch-pasting resistance to the heat with lower values having greater thresholds when withstanding the heat; SV reflects the tendency of starch paste to retrograde; P_Temp_ can reflect the energy cost required during cooking [35]. The PV, HV, BV, FV and SV ranged from 3813 to 4389 mPa s, from 2056 to 2740 mPa s, from 1448 to 2284 mPa s, from 2761 to 3402 mPa s and from 594 to 786 mPa s, respectively, and P_Time_ and P_Temp_ ranged from 4.17 to 4.44 min and from 72.35 to 80.38 °C, respectively, among starches from the developing root tubers of three sweet potato varieties (Table 4). Noda et al. [16] reported that a higher PV is associated with a later harvesting date, P_Temp_ clearly decreases with later harvesting dates, and the other pasting properties are influenced by harvesting dates differently among different sweet potato varieties. The above pasting property parameters were in agreement with the previous reports in sweet potato starch [16]. The pasting properties of starch are influenced by many factors, including starch size, components and crystalline structure [13].

### 3.8. Digestion of Starch

Starch is enzymatically digested into glucose in the digestive system and is utilized as a key source of energy for physiologic processes in the human body. However, some starches and starch degradation products cannot be digested by the small intestine in healthy people. Instead, they reach the colon and are fermented to variable degrees by gut microbes. These starches and starch degradation products are defined as resistant starch (RS) [36]. In the literature, starch is divided into digestible starch and RS, with the latter being the percentage of undigested starch following the incubation of PPA and amyloglucosidase for 16 h at 37 °C [37,38]. In fact, it is more scientific to divide starch into rapidly digestible starch (RDS) (being hydrolyzed within 20 min), slowly digestible starch (SDS) (being hydrolyzed within 20−120 min) and RS (not being hydrolyzed within 120 min) based on the time taken for digestion via amylase in the human body. RDS consists mainly of amorphous and dispersed starch, and SDS can be completely digested in the small intestine, but is digested slowly [36,39]. In this study, the glucose released from native and gelatinized starch by both PPA and AAG was measured at 20 and 120 min, and the RDS, SDS and RS were calculated (Table 5). The native starches from the developing root tubers of three sweet potato varieties had RDS values from 2.7% to 6.8%, SDS from 10.7% to 19.6% and RS from 73.6% to 86.3%, and the gelatinized starch had RDS values from 74.2% to 84.6%, SDS from 0.9% to 3.7% and RS from 14.2% to 24.3%. Native starches contain semicrystalline granule structures, displaying differences in shape, size, starch component and crystalline structure [1,29]. Granules can swell, meaning the crystallinity is disrupted during starch gelatinization, and starch components play an important role in the digestion of gelatinized starch [13]. In this study, the digestion of starch had slight differences among root tubers at different DAPs, but NZS1 starch had lower RDS and higher RS values than SS16 and SS28 starches.

### 3.9. Principal Component Analysis (PCA) of Starch

**Figure 10:**
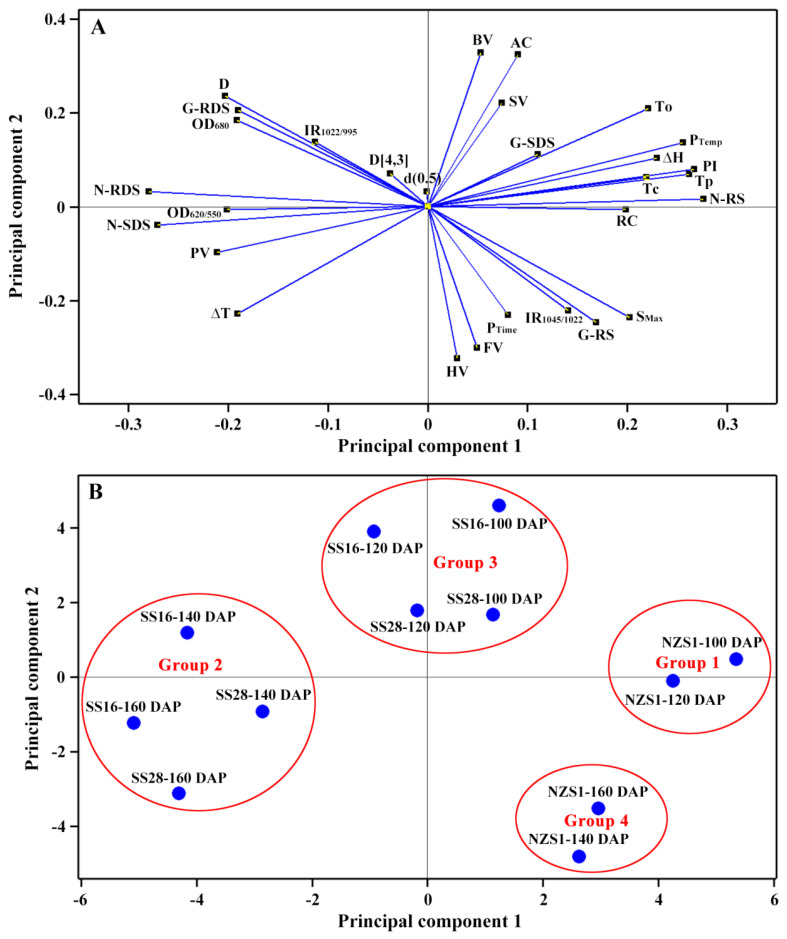
PCA of the physicochemical properties of starches from the developing root tubers of sweet potatoes. (**A**), loading plot of the PCA of starch property parameters; (**B**), score plot of the PCA of starches from the developing root tubers of three sweet potato varieties at 100, 120, 140 and 160 DAP.

In order to further reveal the relationships of starch properties and the differences of starches from the developing root tubers of different sweet potato varieties, we carried out PCA based on the above property parameter data (Figure 10). The principal component 1 and 2 could clarify 42.8% and 28.7% of the total variance, respectively. The loading plot of the PCA exhibited that the gelatinization temperature and enthalpy were highly correlated with the relative crystallinity (RC), lamellar peak intensity (PI), pasting temperature (P_Temp_) and resistant starch (RS) content of starch; the peak viscosity (PV), RDS and SDS of native starch was highly correlated and the AC and BV displayed positive correlations (Figure 10A). The score plot of the PCA exhibited that starches were significantly different among developing root tubers and varieties, and could even be divided into four groups (Figure 10B). Group 1 contained NZS1 at 100 and 120 DAP and was located at the far right of the score plot, and Group 2 contained SS16 and SS28 at 140 and 160 DAP and was located at the far left of the score plot, indicating that Group 1 and Group 2 had significantly different physicochemical properties. Interestingly, Group 3 contained SS16 and SS28 at 100 and 120 DAP, and Group 4 contained NZS1 at 140 and 160 DAP. The score plot of the PCA indicated that the harvesting dates of the root tubers played more important roles in terms of starch properties than the variety type.

## 4. Conclusions

The starches of three sweet potato varieties were isolated from developing root tubers at 100, 120, 140 and 160 DAP. Their properties were investigated and compared. Though the starches from different varieties had some differences in terms of physicochemical properties due to their different genetic backgrounds, the harvesting dates had similar effects on the starch properties among the three varieties. Starch shapes had no significant differences among the different harvesting dates, but the granule size was larger in root tubers at 120 and 140 DAP than at 100 and 160 DAP. The amylose content was higher in the root tubers at 100 and 120 DAP than at 140 and 160 DAP. With the development of the root tuber, the B-type crystallinity increased, and the starch changed from C_A_- to C_C_-type starch, and the lamellar peak intensity gradually decreased; however, the short-ranged ordered structure and lamellar thickness displayed no significant changes. The starch gelatinization temperature gradually decreased and the gelatinization temperature range increased in root tubers from 100 to 160 DAP. The pasting and digestion properties of starch had slight differences among root tubers at different harvesting dates. In conclusion, the harvesting date of root tubers influenced starch properties and applications.

## Figures and Tables

**Table 1:** Granule sizes, iodine absorption parameters and amylose contents of starches from the developing root tubers of sweet potatoes.

Starch Sample	Granule Size		Iodine Absorption Parameters		AC (%)
	d(0.5) (μm)	D[4,3] (μm)	OD_680_	OD_620/550_	
NZS1-100 DAP	16.610 ± 0.002 b	16.994 ± 0.003 a	0.305 ± 0.007 bc	1.099 ± 0.002 a	24.3 ± 0.9 de
NZS1-120 DAP	19.439 ± 0.010 j	19.712 ± 0.011 j	0.300 ± 0.008 bc	1.092 ± 0.017 a	23.3 ± 0.5 cd
NZS1-140 DAP	19.022 ± 0.004 i	19.058 ± 0.005 h	0.275 ± 0.002 a	1.126 ± 0.014 a	20.1 ± 0.1 a
NZS1-160 DAP	17.671 ± 0.004 e	18.078 ± 0.006 e	0.291 ± 0.004 ab	1.123 ± 0.005 a	22.0 ± 0.6 bc
SS16-100 DAP	16.256 ± 0.006 a	17.215 ± 0.006 b	0.328 ± 0.008 d	1.140 ± 0.004 a	26.3 ± 0.5 f
SS16-120 DAP	20.475 ± 0.015 l	21.093 ± 0.014 l	0.322 ± 0.003 cd	1.139 ± 0.018 a	25.4 ± 0.5 ef
SS16-140 DAP	18.541 ± 0.007 h	19.470 ± 0.014 i	0.301 ± 0.008 bc	1.130 ± 0.010 a	23.3 ± 0.5 cd
SS16-160 DAP	17.301 ± 0.006 d	18.276 ± 0.006 f	0.295 ± 0.002 ab	1.147 ± 0.037 a	21.2 ± 0.5 ab
SS28-100 DAP	17.693 ± 0.003 f	17.840 ± 0.007 d	0.308 ± 0.007 bcd	1.143 ± 0.023 a	24.3 ± 0.5 de
SS28-120 DAP	19.824 ± 0.001 k	19.859 ± 0.001 k	0.304 ± 0.010 bc	1.103 ± 0.020 a	23.8 ± 0.6 de
SS28-140 DAP	18.196 ± 0.006 g	18.447 ± 0.008 g	0.293 ± 0.001 ab	1.142 ± 0.020 a	21.9 ± 0.2 bc
SS28-160 DAP	17.111 ± 0.009 c	17.535 ± 0.010 c	0.273 ± 0.005 a	1.151 ± 0.041 a	20.0 ± 0.3 a
Sig.	0.927	0.759	0.734	0.105	0.819

d(0.5), granule size at which 50% of all the granules by volume are smaller; D[4,3], volume-weighted mean diameter; OD_680_, iodine absorption value at 680 nm; OD_620/550_, iodine absorption value ratio of at 620 and 550 nm; AC, amylose content. Sig.: the significance of normal distribution of the 12 samples using the Shapiro–Wilk test. Data are means ± standard deviations (*n* = 3). The values with different letters in the same column are significantly different (*p* < 0.05).

**Table 2:** Relative crystallinity, IR ratios and lamellar structure parameters of starches from the developing root tubers of sweet potatoes.

Starch Sample	RC (%)	IR Ratios		Lamellar Structure Parameters		
		IR_1045/1022_ (cm^−1^)	IR_1022/995_ (cm^−1^)	PI (a.u.)	S_Max_ (Å^−1^)	D (nm)
NZS1-100 DAP	26.7 ± 0.8 c	0.670 ± 0.015 ab	1.024 ± 0.028 bcd	467.8 ± 3.0 f	0.064 ± 0.000 e	9.88 ± 0.01 c
NZS1-120 DAP	26.7 ± 0.3 c	0.656 ± 0.032 ab	1.044 ± 0.064 cd	429.9 ± 2.6 e	0.064 ± 0.000 f	9.83 ± 0.01 b
NZS1-140 DAP	24.0 ± 0.9 b	0.702 ± 0.011 b	0.914 ± 0.006 a	414.3 ± 0.9 e	0.065 ± 0.000 g	9.74 ± 0.00 a
NZS1-160 DAP	26.6 ± 0.6 c	0.686 ± 0.001 ab	0.958 ± 0.019 abc	394.0 ± 10.0 d	0.064 ± 0.000 g	9.76 ± 0.00 a
SS16-100 DAP	25.4 ± 0.3 bc	0.655 ± 0.009 ab	1.024 ± 0.023 bcd	425.9 ± 3.1 e	0.062 ± 0.000 a	10.10 ± 0.01 g
SS16-120 DAP	25.2 ± 0.8 bc	0.651 ± 0.015 ab	1.023 ± 0.013 bcd	387.2 ± 1.0 d	0.062 ± 0.000 a	10.10 ± 0.01 g
SS16-140 DAP	21.6 ± 0.0 a	0.643 ± 0.009 a	1.050 ± 0.015 cd	323.7 ± 8.9 b	0.062 ± 0.000 a	10.08 ± 0.01 g
SS16-160 DAP	24.4 ± 1.2 b	0.655 ± 0.013 ab	1.056 ± 0.010 d	293.8 ± 1.4 a	0.062 ± 0.000 a	10.08 ± 0.02 g
SS28-100 DAP	24.0 ± 0.2 b	0.678 ± 0.012 ab	0.949 ± 0.021 ab	421.5 ± 0.7 e	0.063 ± 0.000 d	9.98 ± 0.00 d
SS28-120 DAP	25.1 ± 0.5 bc	0.668 ± 0.003 ab	0.977 ± 0.005 abcd	390.2 ± 0.6 d	0.063 ± 0.000 bc	10.02 ± 0.01 ef
SS28-140 DAP	23.8 ± 0.3 b	0.663 ± 0.008 ab	1.007 ± 0.004 bcd	362.9 ± 6.0 c	0.063 ± 0.000 b	10.04 ± 0.00 f
SS28-160 DAP	24.7 ± 0.3 bc	0.664 ± 0.007 ab	1.021 ± 0.013 bcd	324.5 ± 2.5 b	0.063 ± 0.000 cd	10.00 ± 0.01 de
Sig.	0.282	0.475	0.184	0.663	0.080	0.058

RC, relative crystallinity; IR_1045/1022_, IR absorption ratio of at 1045 to 1022 cm^−1^; IR_1022/995_, IR absorption ratio of at 1022 to 995 cm^−1^; PI, peak intensity; S_Max_, peak position; D, lamellar distance. Sig.: the significance of normal distribution of the 12 samples using the Shapiro–Wilk test. Data are means ± standard deviations (*n* = 2). The values with different letters in the same column are significantly different (*p* < 0.05).

**Table 3:** Thermal property parameters of starches from the developing root tubers of sweet potatoes.

Starch Sample	To (°C)	Tp (°C)	Tc (°C)	∆T (°C)	∆H (J/g)
NZS1-100 DAP	69.8 ± 0.0 g	75.6 ± 0.1 h	84.1 ± 0.1 d	14.3 ± 0.1 bc	11.84 ± 0.47 d
NZS1-120 DAP	67.7 ± 0.4 f	74.3 ± 0.2 g	81.3 ± 0.4 c	13.6 ± 0.0 ab	11.10 ± 0.11 cd
NZS1-140 DAP	59.9 ± 0.1 d	72.9 ± 0.0 ef	80.4 ± 0.2 bc	20.5 ± 0.1 d	10.53 ± 0.44 bcd
NZS1-160 DAP	59.2 ± 0.2 d	73.7 ± 0.1 fg	80.9 ± 0.1 c	21.7 ± 0.1 d	10.41 ± 0.45 bcd
SS16-100 DAP	68.7 ± 0.1 fg	74.5 ± 0.0 g	81.4 ± 0.1 c	12.8 ± 0.2 a	11.53 ± 0.13 cd
SS16-120 DAP	64.8 ± 0.2 e	72.5 ± 0.1 de	80.1 ± 0.1 abc	15.4 ± 0.4 c	9.88 ± 0.53 abc
SS16-140 DAP	56.6 ± 0.3 c	68.8 ± 0.5 b	80.4 ± 0.5 bc	23.8 ± 0.2 e	9.16 ± 0.54 ab
SS16-160 DAP	53.6 ± 0.3 b	66.0 ± 0.1 a	78.4 ± 1.0 ab	24.8 ± 1.3 e	10.09 ± 0.90 abc
SS28-100 DAP	67.2 ± 0.3 f	72.9 ± 0.2 ef	79.5 ± 0.4 abc	12.3 ± 0.1 a	11.15 ± 0.35 cd
SS28-120 DAP	64.9 ± 0.8 e	71.6 ± 0.1 d	78.4 ± 0.3 ab	13.6 ± 0.5 ab	10.10 ± 0.16 abc
SS28-140 DAP	57.4 ± 0.9 c	70.4 ± 0.6 c	78.7 ± 0.9 ab	21.3 ± 0.0 d	9.85 ± 0.54 abc
SS28-160 DAP	51.3 ± 1.0 a	69.7 ± 0.6 c	78.2 ± 0.9 a	26.9 ± 0.1 f	8.45 ± 0.00 a
Sig.	0.409	0.587	0.199	0.096	0.937

To, gelatinization onset temperature; Tp, gelatinization peak temperature; Tc, gelatinization conclusion temperature; ∆T, gelatinization temperature range (Tc − To); ∆H, gelatinization enthalpy. Sig.: the significance of normal distribution of the 12 samples using the Shapiro–Wilk test. Data are means ± standard deviations (*n* = 3). The values with different letters in the same column are significantly different (*p* < 0.05).

**Table 4:** Pasting property parameters of starches from the developing root tubers of sweet potatoes.

Starch Sample	PV (mPa s)	HV (mPa s)	BV (mPa s)	FV (mPa s)	SV (mPa s)	P_Time_ (min)	P_Temp_ (°C)
NZS1-100 DAP	3813 ± 6 a	2365 ± 17 c	1448 ± 23 a	3030 ± 14 b	665 ± 31 ab	4.37 ± 0.05 cd	80.38 ± 0.04 g
NZS1-120 DAP	3979 ± 29 bc	2401 ± 18 cd	1578 ± 11 bc	3115 ± 9 bc	714 ± 9 bc	4.37 ± 0.05 cd	79.20 ± 0.57 fg
NZS1-140 DAP	4247 ± 16 fg	2740 ± 3 f	1507 ± 13 ab	3402 ± 31 d	662 ± 28 ab	4.40 ± 0.00 d	76.33 ± 0.04 cd
NZS1-160 DAP	4084 ± 4 cd	2464 ± 17 de	1620 ± 13 cd	3090 ± 42 bc	626 ± 25 ab	4.44 ± 0.05 d	77.58 ± 0.53 de
SS16-100 DAP	3850 ± 57 a	2056 ± 45 a	1795 ± 12 f	2842 ± 23 a	786 ± 21 c	4.24 ± 0.05 abc	79.23 ± 0.60 fg
SS16-120 DAP	4348 ± 95 gh	2065 ± 50 ab	2284 ± 45 h	2761 ± 84 a	696 ± 34 abc	4.24 ± 0.05 abc	77.60 ± 0.57 de
SS16-140 DAP	4377 ± 13 h	2105 ± 1 ab	2272 ± 13 h	2790 ± 14 a	685 ± 14 ab	4.33 ± 0.00 bcd	74.43 ± 0.53 b
SS16-160 DAP	4340 ± 24 gh	2407 ± 19 cd	1934 ± 43 g	3000 ± 28 b	594 ± 47 a	4.33 ± 0.00 bcd	72.35 ± 0.00 a
SS28-100 DAP	3874 ± 18 ab	2143 ± 18 ab	1731 ± 1 ef	2813 ± 11 a	670 ± 7 ab	4.17 ± 0.05 a	77.98 ± 0.04 ef
SS28-120 DAP	4101 ± 33 de	2150 ± 4 b	1951 ± 37 g	2815 ± 49 a	665 ± 54 ab	4.17 ± 0.05 a	76.00 ± 0.49 c
SS28-140 DAP	4389 ± 7 h	2503 ± 4 e	1887 ± 11 g	3180 ± 1 c	678 ± 4 ab	4.20 ± 0.00 ab	74.78 ± 0.04 b
SS28-160 DAP	4213 ± 24 ef	2529 ± 38 e	1684 ± 62 de	3190 ± 14 c	661 ± 24 ab	4.33 ± 0.00 bcd	73.15 ± 0.07 a
Sig.	0.157	0.266	0.377	0.281	0.207	0.278	0.843

PV: peak viscosity; HV: hot viscosity; BV: breakdown viscosity (PV − HV); FV: final viscosity; SV: setback viscosity (FV − HV); P_Time_: peak time; P_Temp_: pasting temperature. Sig.: the significance of normal distribution of the 12 samples using the Shapiro–Wilk test. Data are means ± standard deviations (*n* = 2). The values with different letters in the same column are significantly different (*p* < 0.05).

**Table 5:** Digestion properties of starches from the developing root tubers of sweet potatoes.

Starch Sample	Native (N) Starch			Gelatinized (G) Starch		
	N-RDS (%)	N-SDS (%)	N-RS (%)	G-RDS (%)	G-SDS (%)	G-RS (%)
NZS1-100 DAP	2.7 ± 0.1 a	11.3 ± 0.1 a	86.1 ± 0.2 d	76.9 ± 1.7 abcd	3.0 ± 0.9 bc	20.0 ± 1.3 bcde
NZS1-120 DAP	3.0 ± 0.2 ab	10.7 ± 0.2 a	86.3 ± 0.4 d	75.2 ± 2.7 ab	2.7 ± 1.1 abc	22.1 ± 2.3 de
NZS1-140 DAP	3.4 ± 0.1 b	11.0 ± 0.4 a	85.6 ± 0.4 d	76.1 ± 2.2 abc	2.2 ± 0.8 abc	21.7 ± 1.4 cde
NZS1-160 DAP	3.2 ± 0.1 b	11.8 ± 1.4 ab	85.0 ± 1.4 d	74.2 ± 2.9 a	1.5 ± 0.4 ab	24.3 ± 2.6 e
SS16-100 DAP	4.9 ± 0.1 cd	12.7 ± 0.4 bc	82.5 ± 0.5 c	84.6 ± 1.4 e	1.2 ± 0.7 ab	14.2 ± 1.7 a
SS16-120 DAP	5.0 ± 0.1 d	13.4 ± 0.1 c	81.7 ± 0.2 c	81.6 ± 1.6 cde	3.7 ± 0.7 c	14.8 ± 1.3 a
SS16-140 DAP	6.6 ± 0.1 f	17.4 ± 0.5 d	76.0 ± 0.6 b	80.6 ± 1.3 bcde	2.3 ± 0.2 abc	17.0 ± 1.4 abcd
SS16-160 DAP	6.8 ± 0.1 f	18.9 ± 0.4 e	74.3 ± 0.5 a	82.6 ± 1.3 de	1.4 ± 1.0 ab	16.0 ± 0.8 ab
SS28-100 DAP	4.5 ± 0.2 c	13.3 ± 0.3 c	82.2 ± 0.5 c	80.8 ± 3.0 bcde	1.4 ± 0.4 ab	17.8 ± 3.2 abcd
SS28-120 DAP	4.8 ± 0.2 cd	13.8 ± 0.1 c	81.4 ± 0.1 c	80.9 ± 2.2 cde	2.2 ± 0.7 abc	16.9 ± 1.5 abc
SS28-140 DAP	6.2 ± 0.1 e	17.1 ± 0.5 d	76.7 ± 0.6 b	80.7 ± 1.9 bcde	1.3 ± 0.6 ab	18.0 ± 2.2 abcd
SS28-160 DAP	6.8 ± 0.3 f	19.6 ± 0.3 e	73.6 ± 0.6 a	81.4 ± 0.9 cde	0.9 ± 0.3 a	17.7 ± 1.2 abcd
Sig.	0.175	0.098	0.113	0.189	0.344	0.541

RDS, rapidly digestible starch; SDS, slowly digestible starch; RS, resistant starch; N-, native starch; G-, gelatinized starch. Sig.: the significance of normal distribution of the 12 samples using the Shapiro–Wilk test. Data are means ± standard deviations (*n* = 3). The values with different letters in the same column are significantly different (*p* < 0.05).

## Data Availability

The original contributions presented in the study are included in the article, further inquiries can be directed to the corresponding author.

## References

[1] Emmambux M.N., Taylor J.R.N. (2013). Morphology, physical, chemical, and functional properties of starches from cereals, legumes, and tubers cultivated in Africa: A review. Starch.

[2] Li M., Daygon V.D., Solah V., Dhital S. (2021). Starch granule size: Does it matter?. Crit. Rev. Food Sci. Nutr..

[3] Seung D. (2020). Amylose in starch: Towards an understanding of biosynthesis, structure and function. New Phytol..

[4] Nakamura Y., Kainuma K. (2022). On the cluster structure of amylopectin. Plant Mol. Biol..

[5] Magallanes-Cruz P.A., Duque-Buitrago L.F., Martínez-Ruiz N.D. (2023). Native and modified starches from underutilized seeds: Characteristics, functional properties and potential applications. Food Res. Int..

[6] Kringel D.H., Halal S.L.M.E., Zavareze E.D., Dias A.R.G. (2020). Methods for the extraction of roots, tubers, pulses, pseudocereals, and other unconventional starches sources: A review. Starch.

[7] Tagliapietra B.L., Felisberto M.H.F., Sanches E.A., Campelo P.H., Clerici M.T.P.S. (2021). Non-conventional starch sources. Curr. Opin. Food Sci..

[8] Ren Y., Guo K., Zhang B., Wei C. (2020). Comparison of physicochemical properties of very small granule starches from endosperms of dicotyledon plants. Int. J. Biol. Macromol..

[9] Ren Y., Wei Q., Lin L., Shi L., Cui Z., Li Y., Huang C., Wei C. (2021). Physicochemical properties of a new starch from ramie (*Boehmeria nivea*) root. Int. J. Biol. Macromol..

[10] Ren Y., Wei Q., Chen H., Shi L., Sheng W., Zhang Z., Li Y., Huang C., Wei C. (2021). Characterization of underutilized root starches from eight varieties of ramie (*Boehmeria nivea*) grown in China. Int. J. Biol. Macromol..

[11] Alam M.K. (2021). A comprehensive review of sweet potato (*Ipomoea batatas* [L.] Lam): Revisiting the associated health benefits. Trends Food Sci. Technol..

[12] Al-Maqtari Q.A., Li B., He H.J., Mahdi A.A., Al-Ansi W., Saeed A. (2024). An overview of the isolation, modification, physicochemical properties, and applications of sweet potato starch. Food Bioprocess Technol..

[13] Guo K., Liu T., Xu A., Zhang L., Bian X., Wei C. (2019). Structural and functional properties of starches from root tubers of white, yellow, and purple sweet potatoes. Food Hydrocoll..

[14] Song H.G., Choi I., Lee J.S., Chung M.N., Yoon C.S., Han J. (2021). Comparative study on physicochemical properties of starch films prepared from five sweet potato (*Ipomoea batatas*) cultivars. Int. J. Biol. Macromol..

[15] Truong V.D., Avula R.Y., Pecota K.V., Yencho G.C., Siddiq M., Uebersax M.A. (2018). Sweetpotato production, processing, and nutritional quality. Handbook of Vegetables and Vegetable Processing.

[16] Noda T., Takahata Y., Sato T., Ikoma H., Mochida H. (1997). Combined effects of planting and harvesting dates on starch properties of sweet potato roots. Carbohydr. Polym..

[17] Noda T., Kobayashi T., Suda I. (2001). Effect of soil temperature on starch properties of sweet potatoes. Carbohydr. Polym..

[18] Genkina N.K., Noda T., Koltisheva G.I., Wasserman L.A., Tester R.F., Yuryev V.P. (2003). Effects of growth temperature on some structural properties of crystalline lamellae in starches extracted from sweet potatoes (*Sunnyred* and *Ayamurasaki*). Starch.

[19] Genkina N.K., Wasserman L.A., Noda T., Tester R.F., Yuryev V.P. (2004). Effects of annealing on the polymorphic structure of starches from sweet potatoes (*Ayamurasaki* and *Sunnyred* cultivars) grown at various soil temperatures. Carbohydr. Res..

[20] Zhu F., Yang X., Cai Y.Z., Bertoft E., Corke H. (2011). Physicochemical properties of sweetpotato starch. Starch.

[21] Wang H.L., Yang Q.H., Ferdinand U., Gong X.W., Qu Y., Gao W.C., Ivanistau A., Feng B.L., Liu M.H. (2020). Isolation and characterization of starch from light yellow, orange, and purple sweet potatoes. Int. J. Biol. Macromol..

[22] Guo K., Zhang L., Bian X., Cao Q., Wei C. (2020). A-, B-and C-type starch granules coexist in root tuber of sweet potato. Food Hydrocoll..

[23] Guo K., Lin L., Li E., Zhong Y., Petersen B.L., Blennow A., Bian X., Wei C. (2022). Effects of growth temperature on multi-scale structure of root tuber starch in sweet potato. Carbohydr. Polym..

[24] Ye F.Y., Li J.F., Zhao G.H. (2020). Physicochemical properties of different-sized fractions of sweet potato starch and their contributions to the quality of sweet potato starch. Food Hydrocoll..

[25] Li Y., Zhao L., Shi L., Lin L., Cao Q., Wei C. (2022). Sizes, components, crystalline structure, and thermal properties of starches from sweet potato varieties originating from different countries. Molecules.

[26] Silva G.D.P.E., Bento J.A.C., Ribeiro G.O., Liao L.M., Soares M.S., Caliar M. (2021). Application potential and technological properties of colored sweet potato starches. Starch.

[27] Dos Santos T.P.R., Leonel M., De Oliveira L.A., Fernandes A.M., Leonel S., Da Silva Nunes J.G. (2023). Seasonal variations in the starch properties of sweet potato cultivars. Horticulturae.

[28] Kaufman R.C., Wilson J.D., Bean S.R., Herald T.J., Shi Y.C. (2015). Development of a 96-well plate iodine binding assay for amylose content determination. Carbohydr. Polym..

[29] Guo Z., Jia X., Zhao B., Zeng S., Xiao J., Zheng B. (2017). C-type starches and their derivatives: Structure and function. Ann. N. Y. Acad. Sci..

[30] He W., Wei C. (2017). Progress in C-type starches from different plant sources. Food Hydrocoll..

[31] Sevenou O., Hill S.E., Farhat I.A., Mitchell J.R. (2002). Organisation of the external region of the starch granule as determined by infrared spectroscopy. Int. J. Biol. Macromol..

[32] Yuryev V.P., Krivandin A.V., Kiseleva V.I., Wasserman L.A., Genkina N.K., Fornal J., Blaszczak W., Schiraldi A. (2004). Structural parameters of amylopectin clusters and semicrystalline growth rings in wheat starches with different amylose content. Carbohydr. Res..

[33] Fujiwara N., Iii C.H., Carlson R. (2016). A comparison of rapid visco analyser starch pasting curves and test baking for under-and overdosed dry baked mixes. Cereal Food. World.

[34] Wang L.Q., Liu W.J., Xu Y., He Y.Q., Luo L.J., Xing Y.Z., Xu C.G., Zhang Q. (2007). Genetic basis of 17 traits and viscosity parameters characterizing the eating and cooking quality of rice grain. Theor. Appl. Genet..

[35] Xu A., Guo K., Liu T., Bian X., Zhang L., Wei C. (2018). Effects of different isolation media on structural and functional properties of starches from root tubers of purple, yellow and white sweet potatoes. Molecules.

[36] Wang Z., Wang S., Xu Q., Kong Q., Li F., Lu L., Xu Y., Wei Y. (2023). Synthesis and functions of resistant starch. Adv. Nut..

[37] Wang A., Jing Y., Cheng Q., Zhou H., Wang L., Gong W., Kou L., Liu G., Meng X., Chen M. (2023). Loss of function of SSIIIa and SSIIIb coordinately confers high RS content in cooked rice. Proc. Natl. Acad. Sci. USA.

[38] Huang L., Xiao Y., Zhao W., Rao Y., Shen H., Gu Z., Fan X., Li Q., Zhang C., Liu Q. (2024). Creating high-resistant starch rice by simultaneous editing of *SS3a* and *SS3b*. Plant Biotechnol. J..

[39] Sajilata M.G., Singhal R.S., Kulkarni P.R. (2006). Resistant starch—A review. Compr. Rev. Food Sci. Food Saf..

